# Supplementary material for: Effects of Ciprofloxacin Alone or in Mixture with Sulfamethoxazole on the Efficiency of Anaerobic Digestion and Its Microbial Community
Source: Antibiotics (Basel). 2022 Aug 17;11(8):1111. doi: 10.3390/antibiotics11081111 (PMC9404932; doi:10.3390/antibiotics11081111)
Supplement: Supplementary file 1 [file antibiotics-11-01111-s001.zip › antibiotics-1825556-supplementary.pdf]

antibiotics

Supplemental Materials

Effects of Ciprofloxacin Alone or in Mixture with Sulfamethoxazole on the Efficiency of Anaerobic Digestion and Its Microbial Community

Valentina Mazzurco Miritana [1,2], Luisa Patrolecco [3,*], Anna Barra Caracciolo [2,*], Andrea Visca [2], Flavia Piccinini [1], Antonella Signorini [1], Silvia Rosa [1], Paola Grenni [2], Gian Luigi Garbini [2], Francesca Spataro [3], Jasmin Rauseo [3] and Giulia Massini [1,2]

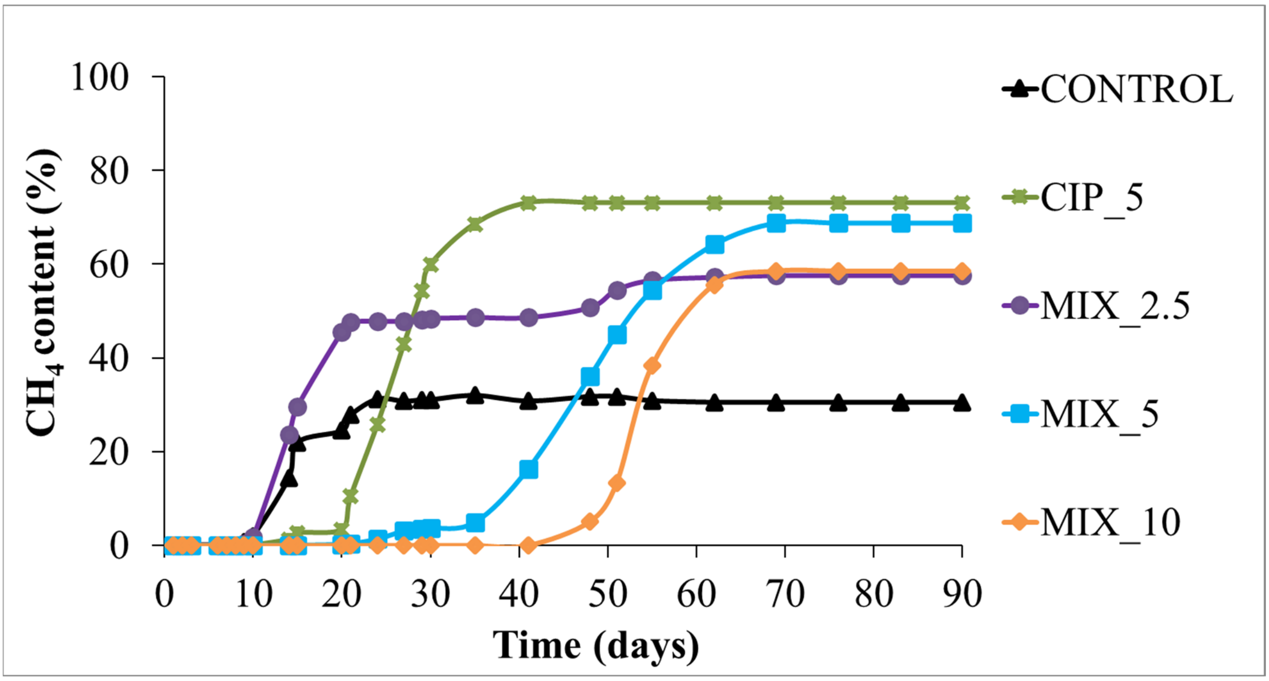

Figure S1. CH_4 content (%) in biogas produced over time by different experimental conditions.
